# Supplementary material for: Molecular basis for dyneinopathies reveals insight into dynein regulation and dysfunction
Source: eLife. 2019 Jul 31;8:e47246. doi: 10.7554/eLife.47246 (PMC6733598; doi:10.7554/eLife.47246)
Supplement: Supplementary file 1. [file elife-47246-supp1.docx]

**Supplementary File 1.** Strains used throughout this study.

| Strain number | Genotype | Yeast background | Source |
| --- | --- | --- | --- |
| SMY1008 | Mata *GAL1p:ZZ-TEV-6HIS-GFP-3HA-GST-dyn1_331_-HaloTag::KAN^R^ prb1∆ pep4∆::HIS5 his3-11,15 ura3-52 leu2-3,112 ade2-1 trp-1* | W303 | Ref^71^ |
| SMY1010 | Mata *DYN1-3GFP::TRP1 TUB1+3'UTR::HPH::HIS3p:mRuby2-TUB1 ura3-52 lys2-801 leu2-∆1 his3-∆200 trp1-∆63* | YEF473 | This study |
| SMY1066 | Mata *dyn1^E109I^-3GFP::TRP1 TUB1+3'UTR::HPH::HIS3p:mRuby2-TUB1 ura3-52 lys2-801 leu2-∆1 his3-∆200 trp1-∆63* | YEF473 | This study |
| SMY1068 | Mata *dyn1^L213I^-3GFP::TRP1 TUB1+3'UTR::HPH::HIS3p:mRuby2-TUB1 ura3-52 lys2-801 leu2-∆1 his3-∆200 trp1-∆63* | YEF473 | This study |
| SMY1073 | Mata *dyn1^W612C^-3GFP::TRP1 TUB1+3'UTR::HPH::HIS3p:mRuby2-TUB1 ura3-52 lys2-801 leu2-∆1 his3-∆200 trp1-∆63* | YEF473 | This study |
| SMY1074 | Mata *dyn1^W612C^-3GFP::TRP1 TUB1+3'UTR::HPH::HIS3p:mRuby2-TUB1 ura3-52 lys2-801 leu2-∆1 his3-∆200 trp1-∆63* | YEF473 | This study |
| SMY1089 | Mata *dyn1^K540C^-3GFP::TRP1 TUB1+3'UTR::HPH::HIS3p:mRuby2-TUB1 ura3-52 lys2-801 leu2-∆1 his3-∆200 trp1-∆63* | YEF473 | This study |
| SMY1090 | Mata *dyn1^K3160Q^-3GFP::TRP1 TUB1+3'UTR::HPH::HIS3p:mRuby2-TUB1 ura3-52 lys2-801 leu2-∆1 his3-∆200 trp1-∆63* | YEF473 | This study |
| SMY1105 | Mata *dyn1^D2439K^-3GFP::TRP1 TUB1+3'UTR::HPH::HIS3p:mRuby2-TUB1 ura3-52 lys2-801 leu2-∆1 his3-∆200 trp1-∆63* | YEF473 | This study |
| SMY1107 | Mata *dyn1^H3639P^-3GFP::TRP1 TUB1+3'UTR::HPH::HIS3p:mRuby2-TUB1 ura3-52 lys2-801 leu2-∆1 his3-∆200 trp1-∆63* | YEF473 | This study |
| SMY1111 | Mata *dyn1^E545V^-3GFP::TRP1 TUB1+3'UTR::HPH::HIS3p:mRuby2-TUB1 ura3-52 lys2-801 leu2-∆1 his3-∆200 trp1-∆63* | YEF473 | This study |
| SMY1112 | Mata *dyn1^E545V^-3GFP::TRP1 TUB1+3'UTR::HPH::HIS3p:mRuby2-TUB1 ura3-52 lys2-801 leu2-∆1 his3-∆200 trp1-∆63* | YEF473 | This study |
| SMY1113 | Mata *dyn1^I554M^-3GFP::TRP1 TUB1+3'UTR::HPH::HIS3p:mRuby2-TUB1 ura3-52 lys2-801 leu2-∆1 his3-∆200 trp1-∆63* | YEF473 | This study |
| SMY1116 | Mata *dyn1^K1475Q^-3GFP::TRP1 TUB1+3'UTR::HPH::HIS3p:mRuby2-TUB1 ura3-52 lys2-801 leu2-∆1 his3-∆200 trp1-∆63* | YEF473 | This study |
| SMY1117 | Mata *dyn1^L2557M^-3GFP::TRP1 TUB1+3'UTR::HPH::HIS3p:mRuby2-TUB1 ura3-52 lys2-801 leu2-∆1 his3-∆200 trp1-∆63* | YEF473 | This study |
| SMY1119 | Mata *dyn1^R3152N^-3GFP::TRP1 TUB1+3'UTR::HPH::HIS3p:mRuby2-TUB1 ura3-52 lys2-801 leu2-∆1 his3-∆200 trp1-∆63* | YEF473 | This study |
| SMY1160 | Matα *DYN1-3GFP::TRP1 kar9∆::KAN^R^ GFP-TUB1::LEU2 ura3-52 lys2- 801 leu2-∆1 his3-∆200 trp1-∆63* | YEF473 | This study |
| SMY1161 | Mata *Dyn1-3GFP::TRP1 kar9∆::KAN^R^ GFP-TUB1::LEU2 ura3-52 lys2- 801 leu2-∆1 his3-∆200 trp1-∆63* | YEF473 | This study |
| SMY1179 | Mata *dyn1^R3201N^-3GFP::TRP1 TUB1+3'UTR::HPH::HIS3p:mRuby2-TUB1 ura3-52 lys2-801 leu2-∆1 his3-∆200 trp1-∆63* | YEF473 | This study |
| SMY1185 | Mata *Dyn1-3GFP::TRP1 NUP133-3mCherry::URA3 kar9∆::KAN^R^ GFP-TUB1::LEU2 ura3-52 lys2- 801 leu2-∆1 his3-∆200 trp1-∆63* | YEF473 | This study |
| SMY1186 | *Mata Dyn1-3GFP::TRP1 NUP133-3mCherry::URA3 kar9∆::KAN^R^ GFP-TUB1::LEU2 ura3-52 lys2- 801 leu2-∆1 his3-∆200 trp1-∆63* | YEF473 | This study |
| SMY1196 | *Matα dyn1^E545V^-3GFP::TRP1 NUP133-3mCherry::URA3 kar9∆::KAN^R^ GFP-TUB1::LEU2 ura3-52 lys2- 801 leu2-∆1 his3-∆200 trp1-∆63* | YEF473 | This study |
| SMY1198 | *Mata dyn1^E109I^-3GFP::TRP1 NUP133-3mCherry::URA3 kar9∆::KAN^R^ GFP-TUB1::LEU2 ura3-52 lys2- 801 leu2-∆1 his3-∆200 trp1-∆63* | YEF473 | This study |
| SMY1200 | Matα *dyn1^L2557M^-3GFP::TRP1 NUP133-3mCherry::URA3 kar9∆::KAN^R^ GFP-TUB1::LEU2 ura3-52 lys2- 801 leu2-∆1 his3-∆200 trp1-∆63* | YEF473 | This study |
| SMY1201 | Matα *dyn1^L2557M^-3GFP::TRP1 NUP133-3mCherry::URA3 kar9∆::KAN^R^ GFP-TUB1::LEU2 ura3-52 lys2- 801 leu2-∆1 his3-∆200 trp1-∆63* | YEF473 | This study |
| SMY1220 | Mata *dyn1^R2439K^-3GFP::TRP1 NUP133-3mCherry::URA3 kar9∆::KAN^R^ GFP-TUB1::LEU2 ura3-52 lys2- 801 leu2-∆1 his3-∆200 trp1-∆63* | YEF473 | This study |
| SMY1222 | Mata *dyn1^K540C^-3GFP::TRP1 NUP133-3mCherry::URA3 kar9∆::KAN^R^ GFP-TUB1::LEU2 ura3-52 lys2- 801 leu2-∆1 his3-∆200 trp1-∆63* | YEF473 | This study |
| SMY1243 | Mata *dyn1^H3639P^-3GFP::TRP1 TUB1+3'UTR::HPH::HIS3p:mRuby2-TUB1 ura3-52 lys2-801 leu2-∆1 his3-∆200 trp1-∆63* | YEF473 | This study |
| SMY1254 | Matα *dyn1^R3152N^-3GFP::TRP1 NUP133-3mCherry::URA3 kar9∆::KAN^R^ GFP-TUB1::LEU2 ura3-52 lys2- 801 leu2-∆1 his3-∆200 trp1-∆63* | YEF473 | This study |
| SMY1266 | Mata *DYN3-13MYC::HPH PAC11-13MYC::TRP ZZ-TEV-3HA-DYN1-HaloTag::KAN^R^ nip100∆ pep4∆::HIS5 prb1∆ his3-11,15 ura3-52 leu2-3,112 ade2-1 trp-1* | W303 | This study |
| SMY1317 | Mata *dyn1^R1852C^-3GFP::TRP1 TUB1+3'UTR::HPH::HIS3p:mRuby2-TUB1 ura3-52 lys2-801 leu2-∆1 his3-∆200 trp1-∆63* | YEF473 | This study |
| SMY1318 | Mata *dyn1^R3201N^-3GFP::TRP1 TUB1+3'UTR::HPH::HIS3p:mRuby2-TUB1 ura3-52 lys2-801 leu2-∆1 his3-∆200 trp1-∆63* | YEF473 | This study |
| SMY1327 | Mata *dyn1^R241L^-3GFP::TRP1 TUB1+3'UTR::HPH::HIS3p:mRuby2-TUB1 ura3-52 lys2-801 leu2-∆1 his3-∆200 trp1-∆63* | YEF473 | This study |
| SMY1351 | Mata *dyn1^R1852C^-3GFP::TRP1 NUP133-3mCherry::URA3 kar9∆::KAN^R^ GFP-TUB1::LEU2 ura3-52 lys2- 801 leu2-∆1 his3-∆200 trp1-∆63* | YEF473 | This study |
| SMY1369 | Mata *dyn1^W3640P^-3GFP::TRP1 TUB1+3'UTR::HPH::HIS3p:mRuby2-TUB1 ura3-52 lys2-801 leu2-∆1 his3-∆200 trp1-∆63* | YEF473 | This study |
| SMY1370 | Mata *dyn1^W3640P^-3GFP::TRP1 TUB1+3'UTR::HPH::HIS3p:mRuby2-TUB1 ura3-52 lys2-801 leu2-∆1 his3-∆200 trp1-∆63* | YEF473 | This study |
| SMY1371 | Mata *dyn1^F3641P^-3GFP::TRP1 TUB1+3'UTR::HPH::HIS3p:mRuby2-TUB1 ura3-52 lys2-801 leu2-∆1 his3-∆200 trp1-∆63* | YEF473 | This study |
| SMY1372 | Mata *dyn1^F3641P^-3GFP::TRP1 TUB1+3'UTR::HPH::HIS3p:mRuby2-TUB1 ura3-52 lys2-801 leu2-∆1 his3-∆200 trp1-∆63* | YEF473 | This study |
| SMY1373 | Mata *dyn1^Y3642P^-3GFP::TRP1 TUB1+3'UTR::HPH::HIS3p:mRuby2-TUB1 ura3-52 lys2-801 leu2-∆1 his3-∆200 trp1-∆63* | YEF473 | This study |
| SMY1374 | Mata *dyn1^I3644P^-3GFP::TRP1 TUB1+3'UTR::HPH::HIS3p:mRuby2-TUB1 ura3-52 lys2-801 leu2-∆1 his3-∆200 trp1-∆63* | YEF473 | This study |
| SMY1381 | Mata *dyn1^N283R^-3GFP::TRP1 TUB1+3'UTR::HPH::HIS3p:mRuby2-TUB1 ura3-52 lys2-801 leu2-∆1 his3-∆200 trp1-∆63* | YEF473 | This study |
| SMY1386 | Mata *dyn1^R3201N^-3GFP::TRP1 NUP133-3mCherry::URA3 kar9∆::KAN^R^ GFP-TUB1::LEU2 ura3-52 lys2- 801 leu2-∆1 his3-∆200 trp1-∆63* | YEF473 | This study |
| SMY1420 | Mata *dyn1^G3643P^-3GFP::TRP1 TUB1+3'UTR::HPH::HIS3p:mRuby2-TUB1 ura3-52 lys2-801 leu2-∆1 his3-∆200 trp1-∆63* | YEF473 | This study |
| SMY1443 | Mata *DYN3-13MYC::HPH PAC11-13MYC::TRP ZZ-TEV-3HA-dyn1^R3201N^-HaloTag::KAN^R^ nip100∆ pep4∆::HIS5 prb1∆ his3-11,15 ura3-52 leu2-3,112 ade2-1 trp-1* | W303 | This study |
| SMY1444 | Mata *DYN3-13MYC::HPH PAC11-13MYC::TRP ZZ-TEV-3HA-dyn1^R3201N^-HaloTag::KAN^R^ nip100∆ pep4∆::HIS5 prb1∆ his3-11,15 ura3-52 leu2-3,112 ade2-1 trp-1* | W303 | This study |
| SMY1445 | Mata *DYN3-13MYC::HPH PAC11-13MYC::TRP ZZ-TEV-3HA-dyn1^D2439K^-HaloTag::KAN^R^ nip100∆ pep4∆::HIS5 prb1∆ his3-11,15 ura3-52 leu2-3,112 ade2-1 trp-1* | W303 | This study |
| SMY1447 | Mata *DYN3-13MYC::HPH PAC11-13MYC::TRP ZZ-TEV-3HA-dyn1^L2557M^-HaloTag::KAN^R^ nip100∆ pep4∆::HIS5 prb1∆ his3-11,15 ura3-52 leu2-3,112 ade2-1 trp-1* | W303 | This study |
| SMY1448 | Mata *DYN3-13MYC::HPH PAC11-13MYC::TRP ZZ-TEV-3HA-dyn1^K1475Q^-HaloTag::KAN^R^ nip100∆ pep4∆::HIS5 prb1∆ his3-11,15 ura3-52 leu2-3,112 ade2-1 trp-1* | W303 | This study |
| SMY1449 | Mata *DYN3-13MYC::HPH PAC11-13MYC::TRP ZZ-TEV-3HA-dyn1^W612C^-HaloTag::KAN^R^ nip100∆ pep4∆::HIS5 prb1∆ his3-11,15 ura3-52 leu2-3,112 ade2-1 trp-1* | W303 | This study |
| SMY1455 | Mata *DYN3-13MYC::HPH PAC11-13MYC::TRP ZZ-TEV-3HA-dyn1^H3639P^-HaloTag::KAN^R^ nip100∆ pep4∆::HIS5 prb1∆ his3-11,15 ura3-52 leu2-3,112 ade2-1 trp-1* | W303 | This study |
| SMY1456 | Mata *DYN3-13MYC::HPH PAC11-13MYC::TRP ZZ-TEV-3HA-dyn1^E545V^-HaloTag::KAN^R^ nip100∆ pep4∆::HIS5 prb1∆ his3-11,15 ura3-52 leu2-3,112 ade2-1 trp-1* | W303 | This study |
| SMY1458 | Matα/Mata *DYN1-3GFP::TRP1/ DYN1-3GFP::TRP1 NUP133-3mCherry::URA3/NUP133-3mCherry::URA3 kar9∆::KAN^R^/kar9∆::KAN^R^ GFP-TUB1::LEU2/GFP-TUB1::LEU2 ura3-52/ura3-52 lys2- 801/lys2- 801 leu2-∆1/leu2-∆1 his3-∆200/his3-∆200 trp1-∆63/trp1-∆63* | YEF473 | This study |
| SMY1481 | Mata *DYN3-13MYC::HPH PAC11-13MYC::TRP ZZ-TEV-3HA-dyn1^R241L^-HaloTag::KAN^R^ nip100∆ pep4∆::HIS5 prb1∆ his3-11,15 ura3-52 leu2-3,112 ade2-1 trp-1* | W303 | This study |
| SMY1507 | Mata *dyn1^C1822S^-3GFP::TRP1 TUB1+3'UTR::HPH::HIS3p:mRuby2-TUB1 ura3-52 lys2-801 leu2-∆1 his3-∆200 trp1-∆63* | YEF473 | This study |
| SMY1508 | Mata *dyn1^R1822S,R1852C^-3GFP::TRP1 TUB1+3'UTR::HPH::HIS3p:mRuby2-TUB1 ura3-52 lys2-801 leu2-∆1 his3-∆200 trp1-∆63* | YEF473 | This study |
| SMY1509 | Mata *dyn1^R1822S,R1852C^-3GFP::TRP1 TUB1+3'UTR::HPH::HIS3p:mRuby2-TUB1 ura3-52 lys2-801 leu2-∆1 his3-∆200 trp1-∆63* | YEF473 | This study |
| SMY1520 | Mata *DYN3-13MYC::HPH PAC11-13MYC::TRP ZZ-TEV-3HA-dyn1^K3160Q^-HaloTag::KAN^R^ nip100∆ pep4∆::HIS5 prb1∆ his3-11,15 ura3-52 leu2-3,112 ade2-1 trp-1* | W303 | This study |
| SMY1532 | Mata *dyn1^C1822S,R1852C^-3GFP::TRP1 NUP133-3mCherry::URA3 kar9∆::KAN^R^ GFP-TUB1::LEU2 ura3-52 lys2- 801 leu2-∆1 his3-∆200 trp1-∆63* | YEF473 | This study |
| SMY1533 | Mata *dyn1^N283R^-3GFP::TRP1 NUP133-3mCherry::URA3 kar9∆::KAN^R^ GFP-TUB1::LEU2 ura3-52 lys2- 801 leu2-∆1 his3-∆200 trp1-∆63* | YEF473 | This study |
| SMY1545 | Mata *DYN3-13MYC::HPH PAC11-13MYC::TRP ZZ-TEV-3HA-dyn1^K540C^-HaloTag::KAN^R^ nip100∆ pep4∆::HIS5 prb1∆ his3-11,15 ura3-52 leu2-3,112 ade2-1 trp-1* | W303 | This study |
| SMY1546 | Mata *DYN3-13MYC::HPH PAC11-13MYC::TRP ZZ-TEV-3HA-dyn1^K540C^-HaloTag::KAN^R^ nip100∆ pep4∆::HIS5 prb1∆ his3-11,15 ura3-52 leu2-3,112 ade2-1 trp-1* | W303 | This study |
| SMY1547 | Mata *DYN3-13MYC::HPH PAC11-13MYC::TRP ZZ-TEV-3HA-dyn1^E545V^-HaloTag::KAN^R^ nip100∆ pep4∆::HIS5 prb1∆ his3-11,15 ura3-52 leu2-3,112 ade2-1 trp-1* | W303 | This study |
| SMY1565 | Matα *dyn1^C1822S,R1852C^-3GFP::TRP1 NUP133-3mCherry::URA3 kar9∆::KAN^R^ GFP-TUB1::LEU2 ura3-52 lys2- 801 leu2-∆1 his3-∆200 trp1-∆63* | YEF473 | This study |
| SMY1585 | Mata *GAL1p:ZZ-TEV-6HIS-GFP-3HA-GST-dyn1_331_^C1822S^-HaloTag::KAN^R^ prb1∆ pep4∆::HIS5 his3-11,15 ura3-52 leu2-3,112 ade2-1 trp-1* | W303 | This study |
| SMY1588 | Mata *GAL1p:ZZ-TEV-6HIS-GFP-3HA-GST-dyn1_331_^R1852C^-HaloTag::KAN^R^ prb1∆ pep4∆::HIS5 his3-11,15 ura3-52 leu2-3,112 ade2-1 trp-1* | W303 | This study |
| SMY1589 | Mata *GAL1p:ZZ-TEV-6HIS-GFP-3HA-GST-dyn1_331_^R1852C^-HaloTag::KAN^R^ prb1∆ pep4∆::HIS5 his3-11,15 ura3-52 leu2-3,112 ade2-1 trp-1* | W303 | This study |
| SMY1591 | Matα *dyn1^H3639P^-3GFP::TRP1 NUP133-3mCherry::URA3 kar9∆::KAN^R^ GFP-TUB1::LEU2 ura3-52 lys2- 801 leu2-∆1 his3-∆200 trp1-∆63* | YEF473 | This study |
| SMY1592 | Matα/Mata *DYN1-3GFP::TRP1/ dyn1∆::HIS3 NUP133-3mCherry::URA3/NUP133-3mCherry::URA3 kar9∆::KAN^R^/kar9∆::KAN^R^ GFP-TUB1::LEU2/GFP-TUB1::LEU2 ura3-52/ura3-52 lys2- 801/lys2- 801 leu2-∆1/leu2-∆1 his3-∆200/his3-∆200 trp1-∆63/trp1-∆63* | YEF473 | This study |
| SMY1627 | Mata *dyn1^R2543K^-3GFP::TRP1 TUB1+3'UTR::HPH::HIS3p:mRuby2-TUB1 ura3-52 lys2-801 leu2-∆1 his3-∆200 trp1-∆63* | YEF473 | This study |
| SMY1628 | Mata *dyn1^R2543K^-3GFP::TRP1 TUB1+3'UTR::HPH::HIS3p:mRuby2-TUB1 ura3-52 lys2-801 leu2-∆1 his3-∆200 trp1-∆63* | YEF473 | This study |
| SMY1651 | Mata *GAL1p:ZZ-TEV-6HIS-GFP-3HA-GST-dyn1_331_^H3639P^-HaloTag::KAN^R^ prb1∆ pep4∆::HIS5 his3-11,15 ura3-52 leu2-3,112 ade2-1 trp-1* | W303 | This study |
| SMY1678 | Matα/Mata *DYN1/ dyn1^E545V^::TRP1 NUP133-3mCherry::URA3/NUP133-3mCherry::URA3 kar9∆::KAN^R^/kar9∆::KAN^R^ GFP-TUB1::LEU2/GFP-TUB1::LEU2 ura3-52/ura3-52 lys2- 801/lys2- 801 leu2-∆1/leu2-∆1 his3-∆200/his3-∆200 trp1-∆63/trp1-∆63* | YEF473 | This study |
| SMY1679 | Matα/Mata *DYN1/ dyn1^H3639P^::TRP1 NUP133-3mCherry::URA3/NUP133-3mCherry::URA3 kar9∆::KAN^R^/kar9∆::KAN^R^ GFP-TUB1::LEU2/GFP-TUB1::LEU2 ura3-52/ura3-52 lys2- 801/lys2- 801 leu2-∆1/leu2-∆1 his3-∆200/his3-∆200 trp1-∆63/trp1-∆63* | YEF473 | This study |
| SMY1697 | Mata *dyn1^C1822S^-3GFP::TRP1 NUP133-3mCherry::URA3 kar9∆::KAN^R^ GFP-TUB1::LEU2 ura3-52 lys2- 801 leu2-∆1 his3-∆200 trp1-∆63* | YEF473 | This study |
| SMY1698 | Mata *GAL1p:ZZ-TEV-6HIS-GFP-3HA-GST-dyn1_331_^C1822S,R1852C^-HaloTag::KAN^R^ prb1∆ pep4∆::HIS5 his3-11,15 ura3-52 leu2-3,112 ade2-1 trp-1* | W303 | This study |
| SMY1699 | Mata *GAL1p:ZZ-TEV-6HIS-GFP-3HA-GST-dyn1_331_^C1822S,R1852C^-HaloTag::KAN^R^ prb1∆ pep4∆::HIS5 his3-11,15 ura3-52 leu2-3,112 ade2-1 trp-1* | W303 | This study |
| SMY1727 | Matα *dyn1^I554M^-3GFP::TRP1 NUP133-3mCherry::URA3 kar9∆::KAN^R^ GFP-TUB1::LEU2 ura3-52 lys2- 801 leu2-∆1 his3-∆200 trp1-∆63* | YEF473 | This study |
| SMY1732 | Mata *dyn1^R2543K^-3GFP::TRP1 NUP133-3mCherry::URA3 kar9∆::KAN^R^ GFP-TUB1::LEU2 ura3-52 lys2- 801 leu2-∆1 his3-∆200 trp1-∆63* | YEF473 | This study |
| SMY1733 | Mata *dyn1^R2543K^-3GFP::TRP1 NUP133-3mCherry::URA3 kar9∆::KAN^R^ GFP-TUB1::LEU2 ura3-52 lys2- 801 leu2-∆1 his3-∆200 trp1-∆63* | YEF473 | This study |
| SMY1740 | Mata *DYN3-13MYC::HPH PAC11-13MYC::TRP ZZ-TEV-3HA-dyn1^I554M^-HaloTag::KAN^R^ nip100∆ pep4∆::HIS5 prb1∆ his3-11,15 ura3-52 leu2-3,112 ade2-1 trp-1* | W303 | This study |
| SMY1744 | Mata *dyn1^R241L^-3GFP::TRP1 NUP133-3mCherry::URA3 kar9∆::KAN^R^ GFP-TUB1::LEU2 ura3-52 lys2- 801 leu2-∆1 his3-∆200 trp1-∆63* | YEF473 | This study |
| SMY1754 | Mata *DYN3-13MYC::HPH PAC11-13MYC::TRP ZZ-TEV-3HA-dyn1^R2543K^-HaloTag::KAN^R^ nip100∆ pep4∆::HIS5 prb1∆ his3-11,15 ura3-52 leu2-3,112 ade2-1 trp-1* | W303 | This study |
| SMY1755 | Mata *DYN3-13MYC::HPH PAC11-13MYC::TRP ZZ-TEV-3HA-dyn1^R2543K^-HaloTag::KAN^R^ nip100∆ pep4∆::HIS5 prb1∆ his3-11,15 ura3-52 leu2-3,112 ade2-1 trp-1* | W303 | This study |
| SMY1756 | Mata *DYN3-13MYC::HPH PAC11-13MYC::TRP ZZ-TEV-3HA-dyn1^R3152N^-HaloTag::KAN^R^ nip100∆ pep4∆::HIS5 prb1∆ his3-11,15 ura3-52 leu2-3,112 ade2-1 trp-1* | W303 | This study |
| SMY1766 | Matα *dyn1^K3160Q^-3GFP::TRP1 NUP133-3mCherry::URA3 kar9∆::KAN^R^ GFP-TUB1::LEU2 ura3-52 lys2- 801 leu2-∆1 his3-∆200 trp1-∆63* | YEF473 | This study |
| SMY1774 | Mata *dyn1^W612C^-3GFP::TRP1 NUP133-3mCherry::URA3 kar9∆::KAN^R^ GFP-TUB1::LEU2 ura3-52 lys2- 801 leu2-∆1 his3-∆200 trp1-∆63* | YEF473 | This study |
| SMY1816 | Mata *dyn1^H3639P,W3640G^-3GFP::TRP1 TUB1+3'UTR::HPH::HIS3p:mRuby2-TUB1 ura3-52 lys2-801 leu2-∆1 his3-∆200 trp1-∆63* | YEF473 | This study |
| SMY1833 | Matα *dyn1^L213I^-3GFP::TRP1 NUP133-3mCherry::URA3 kar9∆::KAN^R^ GFP-TUB1::LEU2 ura3-52 lys2- 801 leu2-∆1 his3-∆200 trp1-∆63* | YEF473 | This study |
| SMY1834 | Mata *dyn1^K1475Q^-3GFP::TRP1 NUP133-3mCherry::URA3 kar9∆::KAN^R^ GFP-TUB1::LEU2 ura3-52 lys2- 801 leu2-∆1 his3-∆200 trp1-∆63* | YEF473 | This study |
| SMY1841 | Mata *dyn1^F3638G,H3639P^-3GFP::TRP1 TUB1+3'UTR::HPH::HIS3p:mRuby2-TUB1 ura3-52 lys2-801 leu2-∆1 his3-∆200 trp1-∆63* | YEF473 | This study |
| SMY1842 | Mata *dyn1^F3638G,H3639P^-3GFP::TRP1 TUB1+3'UTR::HPH::HIS3p:mRuby2-TUB1 ura3-52 lys2-801 leu2-∆1 his3-∆200 trp1-∆63* | YEF473 | This study |
| SMY1857 | Mata *dyn1^R1822S,R1852V^-3GFP::TRP1 TUB1+3'UTR::HPH::HIS3p:mRuby2-TUB1 ura3-52 lys2-801 leu2-∆1 his3-∆200 trp1-∆63* | YEF473 | This study |
| SMY1858 | Mata *dyn1^R1852V^-3GFP::TRP1 TUB1+3'UTR::HPH::HIS3p:mRuby2-TUB1 ura3-52 lys2-801 leu2-∆1 his3-∆200 trp1-∆63* | YEF473 | This study |
| SMY1866 | Mata *dyn1^R1852V^-3GFP::TRP1 NUP133-3mCherry::URA3 kar9∆::KAN^R^ GFP-TUB1::LEU2 ura3-52 lys2- 801 leu2-∆1 his3-∆200 trp1-∆63* | YEF473 | This study |
| SMY1867 | Matα *dyn1^C1822S,R1852V^-3GFP::TRP1 NUP133-3mCherry::URA3 kar9∆::KAN^R^ GFP-TUB1::LEU2 ura3-52 lys2- 801 leu2-∆1 his3-∆200 trp1-∆63* | YEF473 | This study |
| SMY1868 | Mata *DYN3-13MYC::HPH PAC11-13MYC::TRP ZZ-TEV-3HA-dyn1^E109I^-HaloTag::KAN^R^ nip100∆ pep4∆::HIS5 prb1∆ his3-11,15 ura3-52 leu2-3,112 ade2-1 trp-1* | W303 | This study |
| SMY1883 | Mata *DYN3-13MYC::HPH PAC11-13MYC::TRP ZZ-TEV-3HA-dyn1^N283R^-HaloTag::KAN^R^ nip100∆ pep4∆::HIS5 prb1∆ his3-11,15 ura3-52 leu2-3,112 ade2-1 trp-1* | W303 | This study |
| SMY1922 | Mata *dyn1^F3638G,H3639P,W3640G^-3GFP::TRP1 TUB1+3'UTR::HPH::HIS3p:mRuby2-TUB1 ura3-52 lys2-801 leu2-∆1 his3-∆200 trp1-∆63* | YEF473 | This study |
| SMY1923 | Mata *dyn1^F3638G,H3639P,W3640G^-3GFP::TRP1 TUB1+3'UTR::HPH::HIS3p:mRuby2-TUB1 ura3-52 lys2-801 leu2-∆1 his3-∆200 trp1-∆63* | YEF473 | This study |
| SMY1933 | Matα *dyn1^F3638G,H3639P,F3640G^-3GFP::TRP1 NUP133-3mCherry::URA3 kar9∆::KAN^R^ GFP-TUB1::LEU2 ura3-52 lys2- 801 leu2-∆1 his3-∆200 trp1-∆63* | YEF473 | This study |
| SMY1934 | Matα *dyn1^F3638G,H3639P,F3640G^-3GFP::TRP1 NUP133-3mCherry::URA3 kar9∆::KAN^R^ GFP-TUB1::LEU2 ura3-52 lys2- 801 leu2-∆1 his3-∆200 trp1-∆63* | YEF473 | This study |
| SMY1959 | Mata *DYN3-13MYC::HPH PAC11-13MYC::TRP ZZ-TEV-3HA-dyn1^F3638G,H3639P,W3640G^-HaloTag::KAN^R^ nip100∆ pep4∆::HIS5 prb1∆ his3-11,15 ura3-52 leu2-3,112 ade2-1 trp-1* | W303 | This study |
| SMY1960 | Mata *DYN3-13MYC::HPH PAC11-13MYC::TRP ZZ-TEV-3HA- dyn1^F3638G,H3639P,W3640G^-HaloTag::KAN^R^ nip100∆ pep4∆::HIS5 prb1∆ his3-11,15 ura3-52 leu2-3,112 ade2-1 trp-1* | W303 | This study |
| SMY2104 | Mata *DYN3-13MYC::HPH PAC11-13MYC::TRP ZZ-TEV-3HA-dyn1^R1852C^-HaloTag::KAN^R^ nip100∆ pep4∆::HIS5 prb1∆ his3-11,15 ura3-52 leu2-3,112 ade2-1 trp-1* | W303 | This study |
| SMY2129 | Matα *dyn1^C1822A,R1852C^-3GFP::TRP1 NUP133-3mCherry::URA3 kar9∆::KAN^R^ GFP-TUB1::LEU2 ura3-52 lys2- 801 leu2-∆1 his3-∆200 trp1-∆63* | YEF473 | This study |
| SMY2158 | Matα *DYN1-3GFP::TRP1 TUB1+3'UTR::HPH::HIS3p:mRuby2-TUB1 PDR1::pdr1-DBD-CYC8::LEU2 ura3-52 lys2- 801 leu2-∆1 his3-∆200 trp1-∆63* | YEF473 | This study |
| SMY2162 | Mata *dyn1^H3639P^-3GFP::TRP1 TUB1+3'UTR::HPH::HIS3p:mRuby2-TUB1 PDR1::pdr1-DBD-CYC8::LEU2 ura3-52 lys2- 801 leu2-∆1 his3-∆200 trp1-∆63* | YEF473 | This study |
| SMY2455 | Mata *Jnm1-3mCherry::HIS3* *DYN1-3GFP::TRP1* *num1∆::HIS3 ura3-52 lys2-801 leu2-∆1 his3-∆200 trp1-∆63* | YEF473 | This study |
| SMY2456 | Matα *Jnm1-3mCherry::HIS3* *DYN1-3GFP::TRP1* *num1∆::HIS3 ura3-52 lys2-801 leu2-∆1 his3-∆200 trp1-∆63* | YEF473 | This study |
| SMY2457 | Matα *Jnm1-3mCherry::HIS3* *dyn1^K1475Q^-3GFP::TRP1* *num1∆::HIS3 ura3-52 lys2-801 leu2-∆1 his3-∆200 trp1-∆63* | YEF473 | This study |
| SMY2458 | Mata *Jnm1-3mCherry::HIS3* *dyn1^K1475Q^-3GFP::TRP1* *num1∆::HIS3 ura3-52 lys2-801 leu2-∆1 his3-∆200 trp1-∆63* | YEF473 | This study |
